# Body dysmorphic disorder in aesthetic rhinoplasty candidates

**DOI:** 10.12669/pjms.291.2733

**Published:** 2013

**Authors:** Fathololoomi MR, Goljanian Tabrizi A, Fattahi Bafghi A, Noohi SA, Makhdoom A

**Affiliations:** 1 Fathololoomi MR, MD, Associate Professor, ENT Department, Taleghani Hospital, Shaheed Beheshti University of Medical Sciences, Tehran, Iran.; 2 Goljanian Tabrizi A, MD, Assistant Professor, ENT Department, Taleghani Hospital, Shaheed Beheshti University of Medical Sciences, Tehran, Iran.; 3 Fattahi Bafghi A, MD, Associate Professor, ENT Department, Taleghani Hospital, Shaheed Beheshti University of Medical Sciences, Tehran, Iran.; 4 Noohi SA, MD, Assistant Professor, ENT Department, Taleghani Hospital, Shaheed Beheshti University of Medical Sciences, Tehran, Iran.; 5 Makhdoom A, MD, General Practitioner, ENT Department, Taleghani Hospital, Shaheed Beheshti University of Medical Sciences, Tehran, Iran.

**Keywords:** Body Dysmorphic Disorder (BDD), Rhinoplasty, Cosmetic surgery, Psychiatric disorders

## Abstract

***Objective:*** Body Dysmorphic Disorder (BDD) is a psychiatric disorder defined as obsessive ideas about distorted physical appearance, leading to social, professional and personal dysfunction. Many of these patients seek aesthetic surgery and are generally dissatisfied with the outcome of their surgery. In the present study patients coming to the otolaryngology clinic of Thaleghani Hospital in Tehran seeking rhinoplasty were evaluated.

***Methodology:*** Between October 2010 and October 2011, 130 patients seeking rhinoplasty were recruited in a cross-sectional study. These patients were consecutively interviewed in the otolaryngology clinic of Taleghani Hospital, Tehran, Iran. Questionnaires were filled containing demographic data. BDD was evaluated by a separate questionnaire. Hospital Anxiety and Depression Scale (HADS) questionnaire was filled to evaluate depression and anxiety. Data were analyzed by using SPSS software. The frequency and standard deviations were calculated. Differences between groups were evaluated by using the chi-square, and t-tests.

***Results:*** Ninety nine (76.2%) of rhinoplasty candidates were female and thirty one (23.8%) were male. Eighty five (65.4%) were single and thirty eight (29.2%) were married while seven were divorced. About 63.8% were University students or University graduates. Mean age was 26.43±6.29 years old. 41 patients (31.5%) had BDD. Among BDD patients 12 (29.3%) had concurrent depression and 11 (26.8%) had concurrent anxiety. No statistically significant correlation was found between the presence or severity of BDD and age, gender, marital status, level of education and profession.

***Conclusion:*** Considering the high prevalence of Body Dysmorphic Disorder (BDD) among rhinoplasty candidates, psychiatric evaluation is advisable before surgery to avoid unnecessary operations and patient dissatisfaction.

## Introduction

 Aesthetic operations have become very popular over the past two decades.^1^ In 2000 over 1.3 million aesthetic operations were performed in the United States, showing 198% increase compared to 1992.^2^ Rhinoplasty is one of the most popular aesthetic operations.^3^ In recent years the character of applicants for rhinoplasty^4^^,^^5^ and BDD in them^6^^-^^9^ has been considered by researchers.

 In Iran rhinoplasty has become very popular especially among the young people.^8^ Personality disorders and BDD are common among rhinoplasty candidates.^4^^-^^6^^,^^9^ BDD is a psychiatric disorder defined as obsessive ideas about distorted physical appearance leading to social, professional and personal dysfunction. BDD is found in 1-2% of the general population and different parts of the body may be considered distorted by the patient. Rhinoplasty is the most commonly asked aesthetic surgery by these patients.^10^

 Prevalence of BDD among rhinoplasty candidates have been reported to be 3.2% to 16.6%.^11^^-^^13^ Previous studies in Iran have reported a 12.2% to 27.5% prevalence of BDD among rhinoplasty candidates.^6^^,^^8^ The importance of this finding is that patients with BDD will remain unhappy with the outcome of their surgery and will ask for further surgeries creating problems for the surgeon and themselves. Considering the high prevalence of BDD among rhinoplasty candidates, preoperative psychiatric consultation to identify and treat BDD can help the patient and surgeon and will increase eventual patient satisfaction.

 Presence of multiple psychiatric disorders in BDD patients^14^^-^^16^ is another reason to consider preoperative psychiatric consultation. In the present study prevalence of BDD was determined among patients coming to the otolaryngology clinic of Taleghani Hospital in Tehran seeking rhinoplasty.

## Methodology

 Between October 2010 and October 2011, 130 patients seeking rhinoplasty were enrolled in a cross-sectional study. These patients were consecutively interred interviewed in the otolaryngology clinic of Taleghani Hospital by residents of otolaryngology. Demographic data was recorded in a questionnaire. Presence and severity of BDD were evaluated by a BDD questionnaire based on four questions yielding 12 points. Based on this scale, patients getting 0-4 points were classified as mild BDD, 5-8 as moderate BDD and 9-12 as severe BDD.

 Anxiety and depression were evaluated by the Hospital Anxiety and Depression Scale (HADS) consisting of seven questions about anxiety and seven questions about depression, with each question getting 0 to 3 points. Overall, points above 11 were considered positive for anxiety or depression. BDD and HADS questionnaires have been demonstrated to be consistent and reliable.^17^^-^^19^

 Data were analyzed by using SPSS software. The frequency and standard deviations were calculated. Differences between groups were evaluated by using the chi-square, and t-tests.

## Results

 Demographic data are shown in Table-I. Mean age of rhinoplasty candidates was 26.43±6.29. 41 patients (31.5%) had BDD. BDD was mild in 12 patients (29.3%), moderate in 22 patients (53.7%) and severe in 7 patients (17.0%). Out of the 41 patients diagnosed with BDD 12 patients (29.3%) had concurrent depression and 11 patients (26.8%) had concurrent anxiety.

 BDD was seen in 33.3% of women and 25.8% of men seeking rhinoplasty, revealing no statistically significant difference (p>0.05). BDD was seen in 30.6% of single rhinoplasty candidates and 26.3% of married candidates (p>0.05) (Table-II). No statistically significant correlation was found between the presence or severity of BDD and age, gender, marital status, level of education and profession (Table-III). The mean age of all rhinoplasty candidates was 26.43±6.29. In patients with BDD, the mean age was 25.9±6.05 and 26.67±6.42 in non-BDD patients (p>0.05).

## Discussion

 In the present study 31.5% of rhinoplasty candidates had BDD. 70.7% of BDD patients had moderates or severe form of BDD. This result is similar to other studies reporting a 12.2% to 33% prevalence of BDD among rhinoplasty candidates.^6^^,^^8^^,^^9^ This figure was lower in some studies.^7^^,^^20^ Cultural and social factors in different regions and different methods used to diagnose BDD can explain this difference.

**Table-I:** Demographic data in aesthetic rhinoplasty candidates

*Candidate*	*Frequency (n)*	*Percentage (%)*
Gender		
Male	31	23.8
Female	99	76.2
Total	130	100
Marital Status		
Married	38	29.2
Single	85	65.4
Divorced	7	5.4
Total	130	100
Educational level		
Illiterate	6	4.6
Diploma and Less	41	31.5
Above Diploma	83	63.9
Total	130	100
Profession		
Student	69	53.1
Employed Unemployed	31 30	23.8 23.1
Total	130	100

 Studies carried out in Iran^6^^,^^8^ report a higher prevalence of BDD compared to studies performed in other parts of the world.^7^^,^^9^ Majority (76.2%) of rhinoplasty candidates were female in our study. Similar studies carried out in Iran have reported a female predominance among rhinoplasty candidates ranging from 80% to 86%.^6^^,^^8^^,^^21^

**Table-II:** Relationship between BDD in patient’s seeking rhinoplasty & their demographic variables

	*BDD*		*Normal*		*Total*	
	*Frequency (n)*	*Percentage (%)*	*Frequency (n)*	*Percentage (%)*	*Frequency (n)*	*Percentage (%)*
Gender*						
Male	8	25.8	23	74.2	31	100
Female	33	33.3	66	66.7	99	100
Total	41	31.5	89	68.5	130	100
Marital Status						
Single	26	30.6	59	69.4	85	100
Married	10	26.3	28	73.7	38	100
Divorced	5	71.4	2	28.6	7	100
Total	41	31.5	89	68.5	130	100
Educational Level						
Illiterate	1	16.7	5	83.3	6	100
Diploma and less	15	36.6	26	63.4	41	100
Above Diploma	25	30.1	58	69.9	83	100
Total	41	31.5	89	68.5	130	100
Profession						
Student	21	30.4	48	69.6	69	100
Unemployed	9	29.0	22	71.0	31	100
Employed	11	36.7	19	63.3	30	100
Total	41	31.5	89	68.5	130	100

*In all variables P>0.05*

 In 1997, 43% of men and 56% of women, in the United States were unhappy with their physical appearance.^22^ Rhinoplasty has gained greater popularity in Islamic countries over the past few years.^6^^,^^23^ Wearing hijab is an important factor leading to a high number of rhinoplasties performed in Iran.^6^^,^^8^^,^^21^ In the presence of a nasal deformity women feel more distressed compared to men.^24^

**Table-III:** Relationship between severity of BDD in patient seeking rhinoplasty and their demographic variables.

	*Mild*		*Moderate*		*Severe*		*Total*	
	*Frequency (n)*	*Percentage (%)*	*Frequency (n)*	*Percentage (%)*	*Frequency (n)*	*Percentage (%)*	*Frequency (n)*	*Percentage (%)*
Gender*								
Male	3	37.5	4	50.0	1	12.5	8	100
Female	9	27.3	18	54.5	6	18.2	33	100
Total	12	29.3	22	53.7	7	17.0	41	100
Marital Status								
Single	8	30.8	14	53.8	4	15.4	26	100
Married	4	40.0	5	50.0	1	10.0	10	100
Divorced	0	0.0	3	60.0	2	40.0	5	100
Total	12	29.3	22	53.7	7	17.0	41	100
Educational Level								
Illiterate	0	0.0	1	100.0	0	0.0	1	100
Diploma and less	5	33.3	6	40.0	4	26.7	15	100
Above Diploma	7	28.0	15	60.0	3	12.0	25	100
Total	12	29.3	22	53.7	7	17.0	41	100
Profession								
Student	6	28.6	13	61.9	2	9.5	21	100
Unemployed	2	22.2	4	44.5	3	33.3	9	100
Employed	4	36.4	5	45.4	2	18.2	11	100
Total	12	29.3	22	53.7	7	17.0	41	100

*In all variables P>0.05*

 Over the past few years the importance of preoperative psychiatric evaluation in rhinoplasty candidates has been studied.^4^^,^^9^ Patients with psychiatric disorders are more likely to be dissatisfied with the outcome of their surgery^10^^,^^25^, hence the high prevalence of BDD among rhinoplasty candidates in the present study and previous studies in Iran ^6^^,^^8^, necessitates a high degree of vigilance among surgeons and a preoperative psychiatry consultation.

 About 65.4% of our patients were single and 34.6% were married, corresponding to similar studies in Iran.^8^^,^^21^ Using questionnaire and screening methods in rhinoplasty candidates has gained popularity in recent years, to avoid performing unnecessary surgeries in patients with psychiatric disorders.^6^^,^^10^

 In our study, concurrent anxiety was seen in 26.8% of BDD patients and concurrent depression was seen in 29.3% of BDD patients. Alavi M et al reported a 40% prevalence of concurrent anxiety and depression in BDD patients.^8^

 Collecting data via a psychiatric interview, carried out by a psychiatrist will yield more reliable results. In our study we could not collect data via a psychiatric interview and this is a limitation in our study. Future studies should focus on patient satisfaction and comparing post-operative satisfaction among candidates with BDD with candidates without BDD.

## Conclusions

 Our study demonstrates a higher prevalence of BDD in rhinoplasty candidates compared to the general population in Iran and confirms the finding of previous studies. Therefore preoperative psychiatric consultation seems necessary to achieve a satisfactory outcome.
